# Building a prediction model of college students’ sports behavior based on machine learning method: combining the characteristics of sports learning interest and sports autonomy

**DOI:** 10.1038/s41598-023-41496-5

**Published:** 2023-09-20

**Authors:** Haibo Liu, Wenzhi Hou, Iringan Emolyn, Yu Liu

**Affiliations:** 1 Sports Faculty Department, Liaoning University, Shenyang, 110036 China; 2 Basic Teaching Department of Shandong Jiaotong University (Weihai Campus), Weihai, 264209 China; 3 St. Paul University Philippines Graduate School Department, 3500 Tuguegarao, Philippines

**Keywords:** Health care, Physics

## Abstract

College students’ sports behavior is affected by many factors, and sports learning interest and sports autonomy support are potential psychological characteristic factors, which have important influence value on college students’ sports behavior. Machine learning methods are widely used to construct prediction models and show high efficiency. In order to understand the impact of sports learning interest and sports autonomy support on college students’ sports behavior (physical exercise level), the research decided to use the relevant methods of machine learning to build a prediction model, so as to find the internal relationship between them. This paper summarizes the relevant factors that affect college students’ sports behavior (physical exercise level) from two aspects, namely, sports autonomy and sports learning interest, and surveys the demographic and sociological information of college students as a supplement. The research evaluates the level of the prediction model through the construction of the prediction model of the machine learning algorithm and the comparison method, so as to determine the optimal prediction model. The results show that the prediction accuracy of the logistic regression model is 0.7288, the recall rate is 0.7590, and F1 is 0.7397; The prediction accuracy of KNN model is 0.6895, the recall rate is 0.7596, and F1 is 0.7096; The prediction accuracy of naive Bayesian model is 0.7166, the recall rate is 0.6703, and F1 is 0.6864; the prediction accuracy of LDA model is 0.7263, the recall rate is 0.7290, and F1 is 0.7265; The prediction accuracy of the support vector machine model is 0.6563, the recall rate is 0.7700, and F1 is 0.6845; The prediction accuracy of GBDT model is 0.6953, the recall rate is 0.7039, and the F1 score is 0.6989; The prediction accuracy of the decision tree model is 0.6872, the recall rate is 0.6507, and F1 is 0.6672. The logistic regression model performs best in the combination of sports learning interest and motor autonomy support, due to the combination of its linear classification characteristics, better adaptability, high computational efficiency, and better adaptability to feature selection and outlier processing. The conclusion points out that the prediction level of logistic regression model is the highest when combining sports learning interest and sports autonomy support to predict college students’ sports behavior (sports exercise grade), which also provides an important reference for improving college students’ sports behavior (sports exercise grade).

## Introduction

Machine learning algorithm, as a multi-disciplinary interdisciplinary subject, combines computer and human behavior to build models, so as to obtain new prediction mechanism, as an important research field for individuals to acquire knowledge, skills and improve themselves^1^. Machine learning, the core content of AI, has been used to build prediction models in recent years to serve education and medical care^2^. As another evaluation index of college students’ physical quality, sports behavior has important value for the healthy development of college students’ deep absorption. The influencing factors of sports behavior are influenced by potential factors, including interest, autonomy and other factors^3^. Some studies have taken physical health as an important means to predict students’ academic performance and motor skill learning performance, and as an important educational means to interfere with students’ occupation^4,5^. In their research, they used some computer algorithms to establish a prediction model between physical fitness test indicators and academic performance or motor skill performance, so as to find out the factors that affect students’ prediction results based on the prediction results, and adjust and correct the actual problems, so as to make the improvement of students’ academic performance or motor skills more significant^6^. However, the physical health level of college students in China has declined seriously, and many experts are also making efforts to improve the physical health level of college students. The performance of sports behavior is an important indicator to test the physical health level of college students. Ma^7^ noted in her study that:” How to predict sports behavior has attracted attention from all walks of life”. At present, most of the research mainly uses the statistical method of variable relationship analysis, and the prediction model provided for college students is not accurate^8,9^. In order to overcome this difficulty, many people have turned their attention to the dimension of machine learning model. The machine learning model can divide the original data into training and test data. Then the model is fitted and optimized based on the training data to establish the highest-level variable correlation prediction model, and then the test data is used to evaluate the model level, so as to improve the reliability and accuracy of the model^10^. However, the relevant research on observing college students’ sports behavior through the characteristics of potential interest and autonomy has not been paid attention to, and even many studies have only studied the internal relationship under the significant characteristics. There are fewer studies on the use of knowledge in machine learning-related fields to construct and predict college students’ sports behavior. Based on the basic information of college students, this study constructs a prediction model of college students’ sports behavior with their own sports autonomy and sports learning interest as joint potential characteristic variables. Research through the logistic regression model, K neighbor algorithm (KNN), naive Bayes, linear regression algorithm (LDA), support vector machine, gradient promotion decision tree (GBDT), decision tree model prediction model construction and comparison, understand different models on the construction of college students movement behavior accuracy and model overall level, for the improvement of college students' movement behavior and related theory of rich practical basis.

This study is to collect the characteristic data of Liaoning college students’ sports behavior (physical exercise level), sports learning interest, sports autonomy and other characteristics, and establish the optimal machine learning model based on sports learning interest and sports autonomy to predict college students’ sports behavior, with a view to identifying college students with low level sports behavior, actively configuring training means, and improving college students’ sports behavior level. The optimal machine learning model built by the research institute to predict college students’ sports behavior based on sports learning interest and sports autonomy provides an effective basis for intervention to improve college students’ low-level sports behavior.

## Methods and data

### Data source

In order to obtain sufficient data sets, this study distributed and collected questionnaires to college students in Liaoning University by means of questionnaire stars. A total of 1798 college students’ data were obtained. The content of the questionnaire includes basic information, sports behavior (sports exercise scale), sports learning interest (sports learning interest scale) and sports autonomy (sports autonomy support scale).

#### Physical exercise scale

In order to understand the sports behavior of college students, the research adopted the physical exercise grade scale revised by Chinese scholar Liu^11^ to measure the sports behavior of college students. The scale has been widely used in sports behavior measurement with high recognition and good reliability and validity. The scale includes three items: exercise intensity, exercise time and exercise frequency, and uses Likert’s 5-level scoring scale for evaluation. The lowest score is 1, and the highest score is 5. The calculation method is the product of exercise intensity X exercise time X (exercise frequency—1). The score range of physical exercise behavior is 0–100, in which the score of small amounts of exercise is less than 19, the score of medium amounts of exercise is 20–42, and the score of large amounts of exercise is more than 43. The Cronbachα coefficient of test–retest reliability is 0.82, which meets the needs of this study.

#### College students’ sense of sports autonomy support scale

In order to understand college students’ sense of autonomous sports support, the scale of autonomous sports support developed by Fang et al.^12^ was used to evaluate college students’ sense of autonomous sports support. The scale adopts Likert’s 5-grade scoring mode, with a total of 10 questions, all of which are positive scoring. The lowest score is 1, and the highest score is 5.3. Items 7 and 10 are information atmosphere, 1, 2, 5 and 6 are institutional facilities, and 4, 8 and 9 are sports scene scores. The scores of the 10 items are the scores of the sense of motor autonomy support. The total score is divided into high level (score ≥ 37), medium level (28 ≤ score ≤ 37) and low level (score ≤ 28). Cronbach of the scale α the coefficient is 0.89, which meets the research requirements.

#### College students’ interest in sports learning

This study is based on the “Scale of PE Learning Interest of Primary and Secondary School Students” compiled by Chai and Lin^13^, a Chinese scholar, to improve and revise it as a scale for investigating college students’ PE learning interest. The scale is a Likert 5-level scale, with a minimum score of 1 and a maximum score of 5. Among them, questions 7 and 23 are negative scoring, and the rest are positive scoring. The total score of questions 1–7 is situational interest stimulation, questions 8–12 are situational interest maintenance, questions 13–22 are individual interest germination, and questions 23–26 are individual interest maturity. Cronbach of the scale α the coefficient is 0.87, which meets the research requirements.

### Machine learning model construction process

#### Feature selection of machine learning model

The study decided to select the characteristics of machine learning model from the basic situation of students, sports learning interest and sports autonomy support. Basic characteristics include gender, grade, home address, the only child and whether is the class cadre five factors, sports learning interest contains situational interest, interest and individual interest mature four item score, movement autonomous support contains high, medium and low three level characteristics of the corresponding score. As a method of feature selection for machine models, the recursive feature elimination cross-validation method is based on a certain model and recursively removes the least important features until the specified number of features is reached^14^. The features are sorted according to the model coefficients or important attributes of the features, and a small number of redundant or unimportant features are eliminated through circular recursion, and the specified number of features that contribute the most to the model are retained. This process requires the modeler to set a threshold to determine the final number of retained features. Therefore, the size of threshold setting determines the reliability of the model, and recursive feature elimination cross validation is proposed. Recursive feature elimination cross validation method is a feature selection method that combines recursive feature elimination and cross validation elimination. It can automatically retain the best feature combination and avoid the problem of insufficient model accuracy due to manual threshold setting^15^.

#### Machine learning model establishment process

The research adopts Python 3.9.1 version and uses Sklearn library to build a machine learning prediction model for college students’ sports behavior (physical exercise level). The specific process is as follows:

1. Data collection and preprocessing.

The research collects the basic information of college students, sports learning interest (situational interest stimulation, situational interest maintenance, individual interest germination and individual interest maturity), sports autonomy support, sports behavior (physical exercise level) and other data from students in Liaoning University. The study used an electronic questionnaire survey to collect data. Through the official website of the questionnaire star, we set up the basic information about Liaoning University students, the sports learning interest scale, the sports autonomy support scale and the physical exercise rating scale, and distributed it to Liaoning University students in the form of WeChat QR code and observed the data collection in the background, and preprocesses the data. The continuous variables in the real samples in the data are filled with the median, and the discrete variables are filled with the mode^16^. The preprocessing of this data is to adapt to the operation of different machine learning algorithms, and it is also an important step to compare the prediction level of different models. At the initial stage of the study, 1798 original data were obtained. Through comparative analysis of the basic situation of students filling in the questionnaire, the data of students whose answers were uniform were eliminated, so as to ensure the validity of the data obtained. Finally, 1635 limited questionnaires were obtained.

(2) Division of training set and test set.

The research uses the Hold-out method contained in the Sklearn package in python to randomly divide the data set (n = 1635) to determine the training set and test set. On the basis of comprehensive consideration of the overall data, the research takes 90% (n = 1472) of the data set as the training set and 10% (n = 163) of the data set as the test set. Standardize the data so as to prepare for solving the problem of variable conversion between different dimensions^17^. Then we carry out model training for the data set, search for the optimal parameters during the training process, and evaluate the test set after obtaining a good training model. The test set is considered as the detection of location data, so as to verify the reliability and generalization ability of the model. In the process of data set division, the research examined the overall data, and found that the number of college students in Liaoning who were at a low level of sports behavior was relatively large, and the base was too large, so the division ratio of the data set and the test set was adjusted to improve the prediction level of the model.

(3) Machine learning model algorithm test.

The study will study the data set according to the characteristics and advantages of different machine learning algorithms, so as to determine the advantages and disadvantages of different algorithms in building the prediction model of this study. This study decided to train and test the data in the models of logical regression model, KNN, naive Bayes, LDA, support vector machine, GBDT, and decision tree respectively, so as to determine the prediction model of college students’ sports behavior (physical exercise grade) based on machine learning algorithm. At the same time, the study also decided to study and test the pre-selected model to understand the prediction effect of different algorithms.

(4) Feature selection of machine learning model.

The research employed the Recursive Feature Elimination with Cross-Validation (RFECV) method for feature selection in machine learning model building^18^. This method is a wrapper-based feature selection approach that combines Recursive Feature Elimination (RFE) with Cross-Validation (CV) to automatically select the most influential feature subset for model performance^19^.

Initially, the features considered for selection include the subjects' basic characteristics (gender, grade, residential address, only child status, and class cadre role), physical education interest (contextual interest arousal, contextual interest maintenance, individual interest sprouting, and individual interest maturation scores), and sports autonomy support (scores for high, medium, and low levels of support).

Recursive Feature Elimination is a feature selection method based on a specific model that progressively eliminates features with lower importance until the desired feature quantity is achieved. The features are ranked based on model coefficients or importance scores, and in each iteration, a few redundant or less important features are recursively removed, retaining only the specified number of features that contribute the most to the model^20^.

However, RFE requires manual setting of a threshold to determine the final number of retained features, which may lead to either important feature loss or unnecessary feature retention. To address this issue, the research adopted the RFECV method, which combines RFE with cross-validation. This approach automatically selects the best-performing feature subset by evaluating different feature subsets and selecting the one with the highest scores^21^.

Through the RFECV method, the research ensures the automatic preservation of the most contributive feature combination to the model's performance, effectively avoiding accuracy loss that might occur due to manual threshold setting.

(5) Machine learning model training and optimization.

The network search cross-validation method is used to determine the optimal hyperparameter (c = penalty coefficient; g: kernel function parameter). This method first generates a list of possible values of all parameters in the estimation function, and then combines the values in the list to generate a "grid" for the combined results. Each "grid" training model is used to evaluate the performance and optimize the learning algorithm through K-fold cross-validation method^22^. The training of machine learning model is the basis to verify whether the construction model is tenable, and the degree of excellence of the model is adjusted by optimizing parameters to determine the optimal parameters of the model and optimize the model. The sample size of this study was 1635, which was decomposed into 10 data sets, with 9 data as training set and 1 data as validation set, which provides more stable model evaluation results than k = 5, but the computational cost is relatively high. Therefore, the K = 10 in the studies.

(6) Output machine learning model prediction results.

Train the training set, input and compare the prediction results of each model.

(7) Compare the accuracy and reliability of the machine model.

The study will measure the performance of various machine learning models by accuracy, F1 score and ROC curve drop area, and verify the reliability and generalization level of the models.

#### Machine learning model evaluation

The accuracy rate, F1 score, ROC curve sinking area and recall rate were used as an assessment indicator of the machine learning classification model^23^.

Accuracy refers to the ratio of the number of samples correctly classified by the model to the total number of samples, which is used to measure the overall classification performance of the model. It is an intuitive and easily understood indicator for situations where the category distribution is relatively balanced or differences between categories are not obvious. When the category distribution is relatively balanced, the accuracy can provide an intuitive assessment of the overall performance of the model^24^. Its formula is expressed as: (1).

*F1 score* The F1 score takes into account the model precision (Precision) and recall (Recall). Accuracy refers to the proportion of true cases in all samples predicted to be positive cases. Recall refers to the proportion of all true positive cases correctly predicted as positive. The F1 score is the harmonic mean of precision and recall, which has some sensitivity to the model balance between positive and negative cases. Suitable for scenarios with category imbalance or significance for both positive and negative cases, especially in the more critical tasks of positive case prediction, the F1 score can more comprehensively evaluate the model performance^25^. Its formula is expressed as: (2).

Recall refers to the proportion of all true positive cases correctly predicted as positive. Recall focuses on the coverage of the model for positive cases, and is especially suitable for the key tasks of predicting positive cases, such as disease diagnosis. High recall rate means that the model can capture the true positive case better, but may lead to some miscalculation, affecting the accuracy rate^26^. The formula of recall rate is: (3).

ROC curve sinking area: ROC curve takes the true case rate (True Positive Rate, recall rate) and false positive case rate (False Positive Rate, 1-specificity) of the model at different thresholds as coordinates. AUC refers to the area under the ROC curve, reflecting the trade-off between the true and false-positive case rates of the model at different thresholds. The larger the AUC, the better the model performance, which is particularly important for model evaluation in the case of category imbalance, because it is not affected by the threshold and better reflects the overall classification performance of the model^27^.

In the above formula, the results are all between 0 and 1. The closer the result is to 1, the better the performance of the model is. The closer the result is to 0, the worse the performance of the model is

1 $$ ACC = \frac{TP + TN}{{TP + FP + FN + TN}} $$

2 $$ {\text{F}}1Score = \frac{{2 \times \frac{TP}{{TP \times {\text{FP}}}} \times \frac{TP}{{TP + FN}}}}{{\frac{TP}{{TP \times {\text{FP}}}} + \frac{TP}{{TP + FN}}}} $$

3 $$ RECALL = \frac{TP}{{TP + FN}} $$

#### Model determination

**Figure 1:**
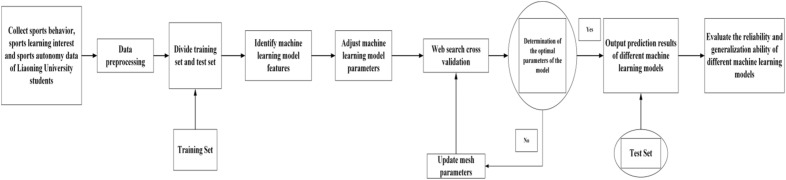
Machine learning model construction process.

In the early stage, after data import and preprocessing, each model imported into machine learning is trained and validated, the results are sorted and compared, and the prediction model used in this study is determined by comparing the evaluation indicators of machine learning model (see Fig. 1). Web search cross validation is detailed in Machine learning model training and optimization.

### Machine learning algorithm theory

#### Logical regression model algorithm

**Figure 2:**
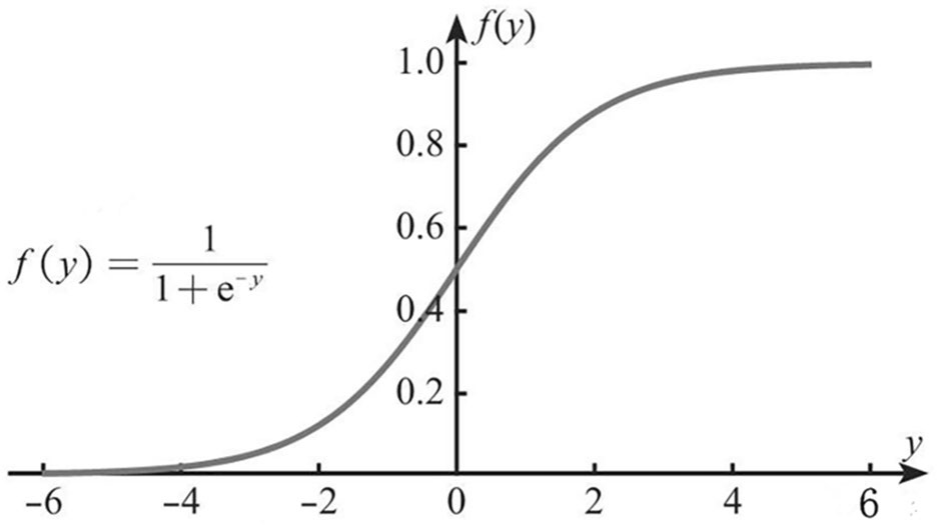
Logistic function.

The logistic regression algorithm uses the logistic function (4) to make the linear regression (${\text{z}} = \mathop w\nolimits^{T} x + b$) fit and approach a decision boundary, and its loss after data classification is minimal. The expression is: (5). When Z > 0 corresponds to the positive sample, the closer it is to positive infinity, the closer L is to 1, and vice versa, the closer it is to 0. The weight w is obtained by logistic regression analysis. The greater the weight w is, the greater the impact it has on the classification results. In logic review, the common loss function is cross entropy. The loss function for n samples is as follows (see Fig. 2):

4 $$ {\text{L}} = \frac{1}{{1 + \mathop {\text{e}}\nolimits^{{ - {\text{z}}}} }} $$

5 $$ \mathop h\nolimits_{{\text{w}}} (x) = \frac{1}{{1 + \mathop e\nolimits^{ - z} }} = \frac{1}{{1 + \mathop e\nolimits^{{ - (\mathop w\nolimits^{T} x + b)}} }} $$

In $y^{\left( i \right)}$ As a sample label value, in binary logical regression $\mathop y\nolimits^{\left( i \right)} \in \{ 0,1\}$. In the whole process of logical regression, finding the optimal decision boundary and making the best classification effect of the model is a process of solving w to minimize the cost loss function (6).

6 $$ \mathop {{\text{cost}}}\nolimits_{{\left( {\text{w}} \right)}} = - \frac{1}{{\text{n}}}[\sum\limits_{i = 1}^{n} ( \mathop y\nolimits^{(i)} \log \mathop h\nolimits_{w} (\mathop x\nolimits^{(i)} ) - (1 - \mathop y\nolimits^{(i)} )\log (1 - \mathop h\nolimits_{w} (\mathop x\nolimits^{(i)} )))] $$

#### GBDT algorithm

It is an iterative decision tree algorithm whose main feature is that it consists of multiple decision trees. Each decision tree is used to fit the prediction residual of the combination of previous decision trees and "correct" the previous model, which is mainly used for regression problems. It uses the characteristics of the training set to divide the data and get the prediction results of each node leaf node. The prediction accuracy is continuously improved through iteration. The algorithm steps are:

1. Initialize the loss function. (7)
2. For the nth iteration, when n ≤ N (N is the base learner), execute (A)—(D), where (n = 1, 2… N). (8)
  - Calculate residual: (9)
  - Fit a regression tree with the residual r to get the leaf node region R of the nth tree, where (j = 1, 2… J).
  - For j = 1, 2… J, the minimum value of linear search loss function:
  - Update f (x): (10)
3. Get the regression number: (11)
4. The model loss function is: (12), $\theta$ is the quantile.
  7 $$ \mathop f\nolimits_{0} (x) = \arg \mathop {\min }\limits_{c} \sum\limits_{i = 1}^{N} {L(\mathop y\nolimits_{i} } ,c) $$
  8 $$ \mathop r\nolimits_{ni} = - \left[ {\frac{{\partial L(\mathop y\nolimits_{i} ,f(\mathop x\nolimits_{i} ))}}{{{\partial }f(\mathop x\nolimits_{i} )}}} \right]f(x) = \mathop f\nolimits_{n - 1} (x) $$
  9 $$ \mathop c\nolimits_{{{\text{mj}}}} = \arg \frac{\min }{c}\sum\limits_{{\mathop x\nolimits_{i} \in \mathop R\nolimits_{nj} }} {L(\mathop y\nolimits_{i} ,\mathop f\nolimits_{n - 1} (\mathop x\nolimits_{i} ) + c)} $$
  10 $$ \mathop f\nolimits_{{\text{n}}} (x) = \mathop f\nolimits_{n - 1} (x) + \sum\limits_{j = 1}^{J} {\mathop c\nolimits_{nj} } I(x \in \mathop R\nolimits_{nj} ) $$
  11 $$ \mathop f\nolimits_{N} (x) = \sum\limits_{n - 1}^{N} {\sum\limits_{j = 1}^{J} {\mathop c\nolimits_{nj} } } I(x \in \mathop R\nolimits_{nj} ) $$
  12 $$ {\text{L}}(y,f(x)) = \sum\nolimits_{y \ge f(x)} {\theta \left| {y - f(x)} \right.} \left| + \right.\sum\nolimits_{y < f(x)} {(1 - \theta )} \left| {y - f(x)\left| {} \right.} \right. $$

#### KNN algorithm

KNN algorithm is derived from Euclidean distance and is a relatively stable and effective algorithm. KNN algorithm is characterized by simple and effective classification model, and its implementation steps are as follows:

1. Data source and processing and feature selection.
2. Scan and unify the vector of each training text in the feature space to determine the weight of each dimension.
3. The cosine distance algorithm is used to calculate the weight values of each text vector and calculate the similarity with each training text. The formula is as follows: (13).

In $\mathop w\nolimits_{{i\mathop k\nolimits^{2} }}$ where $\mathop w\nolimits_{{i\mathop k\nolimits^{2} }}$ is the k-dimensional attribute weight of the text $\mathop d\nolimits_{i}$ and M is the total dimensionality of the text feature vector.

(4) The K training samples closest to the test text are selected according to the descending order of text similarity. According to the similarity between the test text and k nearest neighbor texts and k nearest neighbor categories, calculate the weight of each category that the test text belongs to. The formula is as follows: (14).
  13 $$ Sim(\mathop d\nolimits_{i} ,\mathop d\nolimits_{j} ) = \frac{{\sum\nolimits_{k = 1}^{M} {\mathop w\nolimits_{ik} \times \mathop w\nolimits_{jk} } }}{{\sqrt {(\sum\nolimits_{k = 1}^{M} {\mathop w\nolimits_{{i\mathop k\nolimits^{2} }} } )(\sum\nolimits_{k = j}^{M} {\mathop w\nolimits_{{j\mathop k\nolimits^{2} }} } )} }} $$
  14 $$ \mathop\upmu \nolimits_{j} (x) = \sum\nolimits_{i = 1}^{K} {\mathop\upmu \nolimits_{j} } (\mathop x\nolimits_{i} )sim(X,\mathop X\nolimits_{i} ) $$

In $\mathop\upmu \nolimits_{j} (\mathop X\nolimits_{i} ) \in \{ 0,1\}$,meaning is text $\mathop X\nolimits_{i}$ whether it belongs to category $\mathop c\nolimits_{j}$, ${\text{sim}}\left( {{\text{X,}}\mathop {\text{X}}\nolimits_{{\text{i}}} } \right)$ indicates the similarity between test text and training text. The decision-making method is as follows^28^:

15 $$ \mathop\upmu \nolimits_{1} = \max \mathop\upmu \nolimits_{{\text{j}}} \left( {\text{X}} \right),Decision\quad {\text{X}} \in \mathop {\text{c}}\nolimits_{1} $$

#### Naive Bayesian algorithm

Naive Bayesian algorithm is one of the simple and effective classification models. Its advantages are stable classification efficiency and good performance in small-scale data processing. The point of this algorithm is that the classification efficiency is stable, especially for small-scale data processing. Its disadvantage is the requirement to verify the probabilities first and also the sensitivity of the data expression. The order of work is. Each data sample uses m feature vector X = {$\mathop x\nolimits_{1}$,$\mathop x\nolimits_{2}$…} express. Describing m attributes A_1_, A_2_…, A_n_ N measurements of the sample.

1. In the given assumption, there are n classes, namely C_1_, C_2_… C_n_. When given an unknown number of data samples X, the classification method will predict the category with the highest posterior probability (condition X) for data sample X. The plain Bayesian classification will assign the location samples to various C1. Only if: (16)
2. According to Bayesian theorem (17). Maximize $P(\mathop c\nolimits_{i} |X)$ becomes the maximum posteriori hypothesis.
3. Given a data set with multiple attributes, compute $P(\mathop c\nolimits_{i} |X)$ the cost probability is high, to reduce $P(\mathop c\nolimits_{i} |X)$ expenses, by making independent and simple assumptions of class conditions (18).

It can be judged by the training set. If:

- If Ak were a discrete property (19).

In $\mathop s\nolimits_{ik}$ The value X_k_ with class Ci features in attribute Ak, Si is the number of training samples of Ci.

b. If the attribute of Ak is a continuous attribute, it is generally assumed that the attribute obeys a Gaussian distribution.

Therefore: (20).

Given the values of the training samples of class Ci with attribute Ak. $g(\mathop x\nolimits_{k} ,\mathop u\nolimits_{ci} ,\mathop\upsigma \nolimits_{ci} )$ is the Gaussian density function with the properties of Ak.$\mathop u\nolimits_{ci} ,\mathop\upsigma \nolimits_{ci}$ Mean value and standard deviation respectively.

For location sample X, for each class C_i_, for each class C_i_, calculate $P(X|\mathop c\nolimits_{i} )P(\mathop c\nolimits_{i} )$. Sample X is assigned to class C_i_ and only if $P(X|\mathop c\nolimits_{i} )P(\mathop c\nolimits_{i} ){ > }P(X|\mathop c\nolimits_{j} )P(\mathop c\nolimits_{j} ),1 \le i,j \le m,j \ne i$,in other words, X is assigned to its $P(X|\mathop c\nolimits_{i} )P(\mathop c\nolimits_{i} )$^29^.

16 $$ P(\mathop c\nolimits_{i} |X){ > }P(\mathop c\nolimits_{j} |X),1 \le i,j \le m,j \ne i $$

17 $$ P(\mathop c\nolimits_{i} |X) = \frac{{P(X|\mathop c\nolimits_{i} )P(\mathop c\nolimits_{i} )}}{P(X)} $$

18 $$ P(X|\mathop c\nolimits_{i} ) = \prod\limits_{k = 1}^{n} {p(\mathop x\nolimits_{k} |\mathop c\nolimits_{i} )} ,{\text{probability}}\quad P(\mathop x\nolimits_{1} |\mathop c\nolimits_{i} ),P(\mathop x\nolimits_{2} |\mathop c\nolimits_{i} ),...P(\mathop x\nolimits_{n} |\mathop c\nolimits_{i} ), $$

19 $$ P(\mathop x\nolimits_{k} |\mathop c\nolimits_{i} ) = \mathop s\nolimits_{ik} {/}s $$

20 $$ P(\mathop x\nolimits_{k} |\mathop c\nolimits_{i} ) = g(\mathop x\nolimits_{k} ,\mathop u\nolimits_{{ci,\mathop\upsigma \nolimits_{ci} }} ) = \frac{1}{{\mathop\upsigma \nolimits_{{ci\sqrt {2\uppi } }} }}\mathop e\nolimits^{{ - \frac{{(\mathop x\nolimits_{k} - \mathop u\nolimits_{ci} \mathop )\nolimits^{2} }}{{2\mathop\upsigma \nolimits_{ci} }}}} $$

#### LDA

This algorithm is one of the classic supervised learning methods. The main idea of this algorithm is to project the data in the high-order space onto the position space, so as to ensure that the intra-class variance of each category after projection is minimum and the inter-class mean difference is maximum. The algorithm principle is as follows: Assume that the data set A = {(x_1_, y_1_), (x_2_, y_2_) … (x_N_, y_N_)}, whose X_i_ belongs to R_p_, is a p-dimensional space vector, y_i_ ∈ {C_1_, C_2_ … C_k_}. N_j_ (j = 1, 2… k) is defined as the number of low-j samples, and X_j_ (j = 1, 2… k) is defined as the set of j-class samples. $\mathop {\text{x}}\nolimits_{{{\text{cj}}}}$(j = 1, 2… k) is the mean vector of the class j sample, defined $\sum\nolimits_{{\text{j}}} {\left( {{\text{j}} = 1,2,...k} \right)}$ Its the covariance matrix of the j-type sample. Assume that the projection to the low-dimensional space dimension is q, the corresponding base vector is (W_1_, W_2_… W_q_), and the base vector matrix is W, p × Q matrix, the optimization objective becomes: (21), in (22), $\overline{x}$ is the mean vector. (23) But because $\mathop W\nolimits^{T} \mathop S\nolimits_{b} W$ and $\mathop W\nolimits^{T} \mathop S\nolimits_{w} W$ both are matrices and cannot be optimized as scalar functions. Therefore, the LDA multi-class objective optimization function is: (24). In $\prod\limits_{diag} A$ Is the diagonal product of A, and W is p × Q matrix. The J (W) optimization process is transformed into: (25).

The algorithm flow is as follows:

1. Input data set: A = {(x_1_, y_1_), (x_2_, y_2_) … (x_N_, y_N_)}, whose X_i_ belongs to R_p_, is a p-dimensional space vector, y_i_ ∈ {C_1_, C_2_ … C_k_}, and is reduced to dimension q. Sample set A, after dimension reduction is output.
2. Calculate the intra-class divergence matrix Sw, and then calculate the inter-class divergence matrix Sb.
3. Calculation matrix $$\mathop s\nolimits_{w}^{ - 1} \mathop s\nolimits_{b}^{{}}$$.
4. Calculation $\mathop s\nolimits_{w}^{ - 1} \mathop s\nolimits_{b}^{{}}$ the projection matrix W is obtained from the maximum q eigenvalue and the corresponding q eigenvectors (W1, W2 … Wd).
5. Convert each sample feature Xi in the sample set into a new sample: (26)
6. Output new sample A, = (z_1_, y_1_), (z_2_, y_2_) (z_m_, y_m_).
  21 $$ \frac{{\mathop W\nolimits^{T} \mathop S\nolimits_{b} W}}{{\mathop W\nolimits^{T} \mathop S\nolimits_{w} W}} $$
  22 $$ \mathop S\nolimits_{b} = \sum\limits_{j = 1} {\mathop N\nolimits_{j} } (\overline{{\mathop x\nolimits_{cj} }} - \overline{x} )(\overline{{\mathop x\nolimits_{cj} }} - \overline{x} \mathop )\nolimits^{T} $$
  23 $$ \mathop S\nolimits_{w} = \sum\limits_{j = 1} {\mathop S\nolimits_{wj} } = \sum\limits_{j = 1}^{k} {\sum\limits_{{x \in \mathop x\nolimits_{j} }} {(x - \overline{{\mathop x\nolimits_{cj} }} )} } (x - \overline{{\mathop x\nolimits_{cj} }} \mathop )\nolimits^{T} $$
  24 $$ \mathop {\arg \max }\limits_{{\mathop \omega \limits_{W} }} J(W) = \frac{{\prod\limits_{diag} {\mathop W\nolimits^{T} \mathop S\nolimits_{b} W} }}{{\prod\limits_{diag} {\mathop W\nolimits^{T} \mathop S\nolimits_{w} W} }} $$
  25 $$ {\text{J}}\left( {\text{W}} \right) = \frac{{\prod\limits_{i = 1}^{d} {\mathop w\nolimits_{i}^{T} \mathop S\nolimits_{b} \mathop w\nolimits_{i} } }}{{\prod\limits_{i = 1}^{d} {\mathop w\nolimits_{i}^{T} \mathop S\nolimits_{w} \mathop w\nolimits_{i} } }} = \prod\limits_{i = 1}^{d} {\frac{{\mathop w\nolimits_{i}^{T} \mathop S\nolimits_{b} \mathop w\nolimits_{i} }}{{\mathop w\nolimits_{i}^{T} \mathop S\nolimits_{w} \mathop w\nolimits_{i} }}} $$
  26 $$ \mathop {\text{Z}}\nolimits_{{\text{i}}} = \mathop {\text{W}}\nolimits^{{\text{T}}} \mathop {\text{x}}\nolimits_{{\text{i}}} $$

#### Support vector machine

Support vector machines have greater advantages in solving small sample problems, which can be divided into linear separable and nonlinear separable. This research is mainly based on nonlinear segmentation. The nonlinear segmentation classification model is still based on the linear model: (27). Where a_i_, i = 1, n. N is the optimized solution of the quadratic programming problem. MAXiMize (28), Subject to (29). Set it to sample X_j_, and obtain the support vector: (30).

Make nonlinear transformation to feature X and record it as a new feature $Z = {\varphi }(X)$, the SVM model in the new feature space is: (31).

The corresponding secondary optimization problem is transformed into: (32). MAXiMize (33), and Subject to (34). The SVM equation is transformed into: (34), Use the kernel function to calculate the inner product and record it as (2.3.6–28). The form of support vector machine is transformed into: (35).

Coefficient $\upalpha$ is the solution of the following quadratic optimization problem: MAXiMize (36), and Subject to (37). The coefficient b is solved by support vector: (38). So as to obtain the mapping function $K(\mathop X\nolimits_{i} ,\mathop X\nolimits_{j} )$.

27 $$ f(x) = {\text{sgn}} (WX + {\text{b}}) = {\text{sgn}} (\sum\nolimits_{i = 1}^{N} {\mathop a\nolimits_{i} } \mathop y\nolimits_{i} (\mathop X\nolimits_{i} \cdot X) + b) $$

28 $$ {\text{ Q(}}\upalpha {) = }\sum\nolimits_{i = 1}^{n} {\mathop \upalpha \nolimits_{i} } - \frac{1}{2}\sum\nolimits_{i,j}^{l} {\mathop \upalpha \nolimits_{i} } \mathop \upalpha \nolimits_{j} \mathop y\nolimits_{i} \mathop y\nolimits_{j} \mathop X\nolimits_{i} \mathop X\nolimits_{j} $$

29 $$ \sum\nolimits_{i = 1}^{l} {\mathop \upalpha \nolimits^{i} } \mathop y\nolimits_{i} C \ge \mathop \upalpha \nolimits_{i} \ge 0,i = 1,2,...n $$

30 $$ \mathop y\nolimits_{i} (\sum\nolimits_{i - 1}^{n} {\mathop \upalpha \nolimits_{i} } (\mathop X\nolimits_{i} \cdot \mathop X\nolimits_{j} + b)) - 1 = 0 $$

31 $$ f(x) = {\text{sgn}} (\mathop w\nolimits^{\varphi } Z + b) = {\text{sgn}} (\sum\nolimits_{i = 1}^{N} {\mathop \alpha \nolimits_{i} } \mathop y\nolimits_{i} (\varphi (\mathop X\nolimits_{i} ) \cdot (\varphi (\mathop X\nolimits_{j} ) + b) $$

32 $$ {\text{ Q(}}\upalpha {) = }\sum\nolimits_{i = 1}^{n} {\mathop \upalpha \nolimits_{i} } - \frac{1}{2}\sum\nolimits_{i,j}^{l} {\mathop \upalpha \nolimits_{i} } \mathop \upalpha \nolimits_{j} \mathop y\nolimits_{i} \mathop y\nolimits_{j} ({\varphi }(\mathop X\nolimits_{i} ) \cdot ({\varphi }(\mathop X\nolimits_{j} )) $$

33 $$ \sum\nolimits_{i = 1}^{l} {\mathop \upalpha \nolimits^{i} } \mathop y\nolimits_{i} = 0C \ge \mathop \upalpha \nolimits_{i} \ge 0,i = 1,2,...n $$

34 $$ {\text{K}}(\mathop X\nolimits_{i} ,\mathop X\nolimits_{j} ) = ({\varphi }(\mathop X\nolimits_{i} ) \cdot ({\varphi }(\mathop X\nolimits_{j} )) $$

35 $$ f(x) = {\text{sgn}} (\sum\nolimits_{i = 1}^{N} {\mathop \upalpha \nolimits_{i} } \mathop y\nolimits_{i} K(\mathop X\nolimits_{i} ,\mathop X\nolimits_{j} ) + b) $$

36 $$ {\text{ Q(}}\upalpha {) = }\sum\nolimits_{i = 1}^{n} {\mathop \upalpha \nolimits_{i} } - \frac{1}{2}\sum\nolimits_{i,j}^{l} {\mathop \upalpha \nolimits_{i} } \mathop \upalpha \nolimits_{j} \mathop y\nolimits_{i} \mathop y\nolimits_{j} K(\mathop X\nolimits_{i} ,\mathop X\nolimits_{j} )\mathop \upalpha \nolimits_{i} $$

37 $$ \sum\nolimits_{i = 1}^{l} {\mathop \upalpha \nolimits_{i} } \mathop y\nolimits_{i} = 0C \ge \mathop \upalpha \nolimits_{i} \ge 0,i = 1,2,...n $$

38 $$ \mathop y\nolimits_{{\text{i}}} (\sum\nolimits_{i,j = 1}^{n} {\mathop \upalpha \nolimits_{i} } \mathop y\nolimits_{i} (K(\mathop X\nolimits_{i} ,\mathop X\nolimits_{j} )) + b) - 1 = 0 $$

#### Decision tree algorithm

As a basic classification and regression algorithm, the algorithm has high practicability. The number of decisions usually consists of three parts: feature selection, number of decisions generation, and number of decisions modification. Its advantages are self-learning, good readability and high efficiency. The main process is as follows:

1. Record each node, traverse each feature classification method and find the classification point, classify it into multiple or two nodes, and then continue to split to guide the node to be pure enough.
2. The number of IDE algorithm construction decisions. Calculate the empirical quotient of the dataset (39).

In $\left| {\text{D}} \right|$ Is the number of data set samples, K is the number of target variable categories, $\left. {\mathop C\nolimits_{k} } \right|$ is the number of samples for this classification.

(3) Modify the number of decisions.

By pruning the decision tree obtained from the data set construction, the accuracy of new data classification can be improved^30^.

39 $$ {\text{H(D) = }} - \sum\limits_{k = 1}^{k} {\mathop p\nolimits_{i} } \log (p_{i} ) = - \sum\limits_{k = 1}^{k} {\frac{{\left| {{}C_{k} } \right|}}{\left| D \right|}} \log \frac{{\mathop C\nolimits_{k} }}{{\text{D}}} $$

## Result

### Model feature selection results

This paper uses recursive feature elimination cross-validation methods to determine the number of features and the best feature subset of the model in this construction. The input characteristics of this study mainly include individual specific conditions (gender, grade, home address, only child, class cadre), sports independent support, sports learning interest (situational interest stimulation, situational interest maintenance, personal interest germination and personal interest maturity).

**Figure 3:**
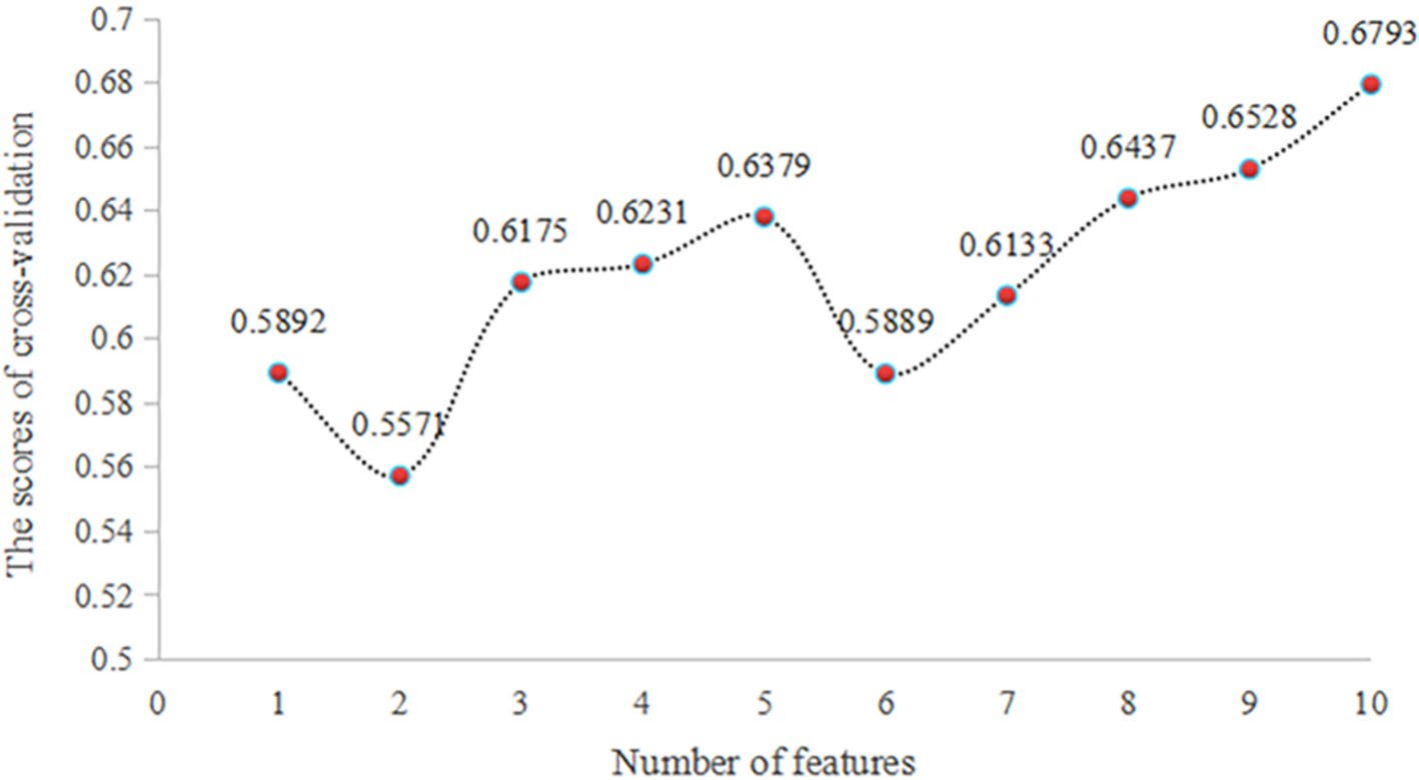
Selects cross-validation scores based on a machine learning feature model combining the characteristics of sports learning interest and sports autonomy.

The subnumber and the best features of machine learning models based on sports learning interest and sports autonomy were selected by RFECV method. As shown in Fig. 3, sports learning interest and sports autonomy prediction machine learning model of sports behavior of 10, the best characteristic subset of gender, grade, family address, whether the only child, class cadres, sports autonomous support, sports learning interest (situational interest stimulation, interest maintenance, personal interest germination and personal interest maturity.

**Figure 4:**
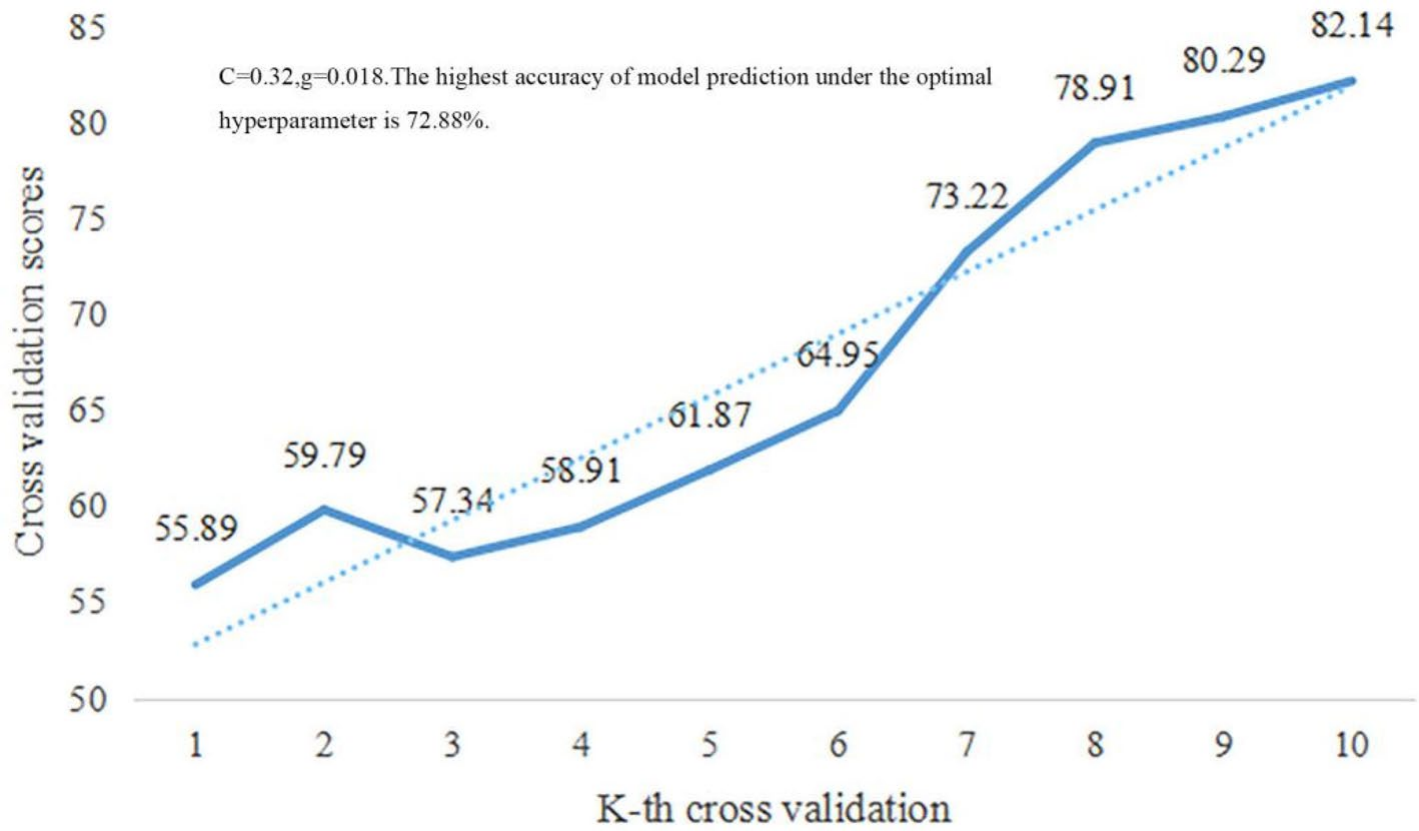
Best hyperparameters of the machine learning feature model combining the characteristics of sports learning interest and sports autonomy. *Note*: c and g are the hyperparameters of the machine learning model. Where C is the penalization coefficient and g is the kernel function parameter.

The best subset of features was included in the machine learning model to predict college students' sports behavior based on sports learning interest and sports autonomy, and the best hyperparameters (c, g) were determined by GridSearchCV. As shown in Fig. 4. Best hyperparameter combination of machine learning model based on sports learning interest and sports autonomy: C = 0.32, g = 0.018. The highest model prediction accuracy under the best hyperparameters was 72.88%.

### Comparison of prediction models under different machine learning algorithms

**Figure 5:**
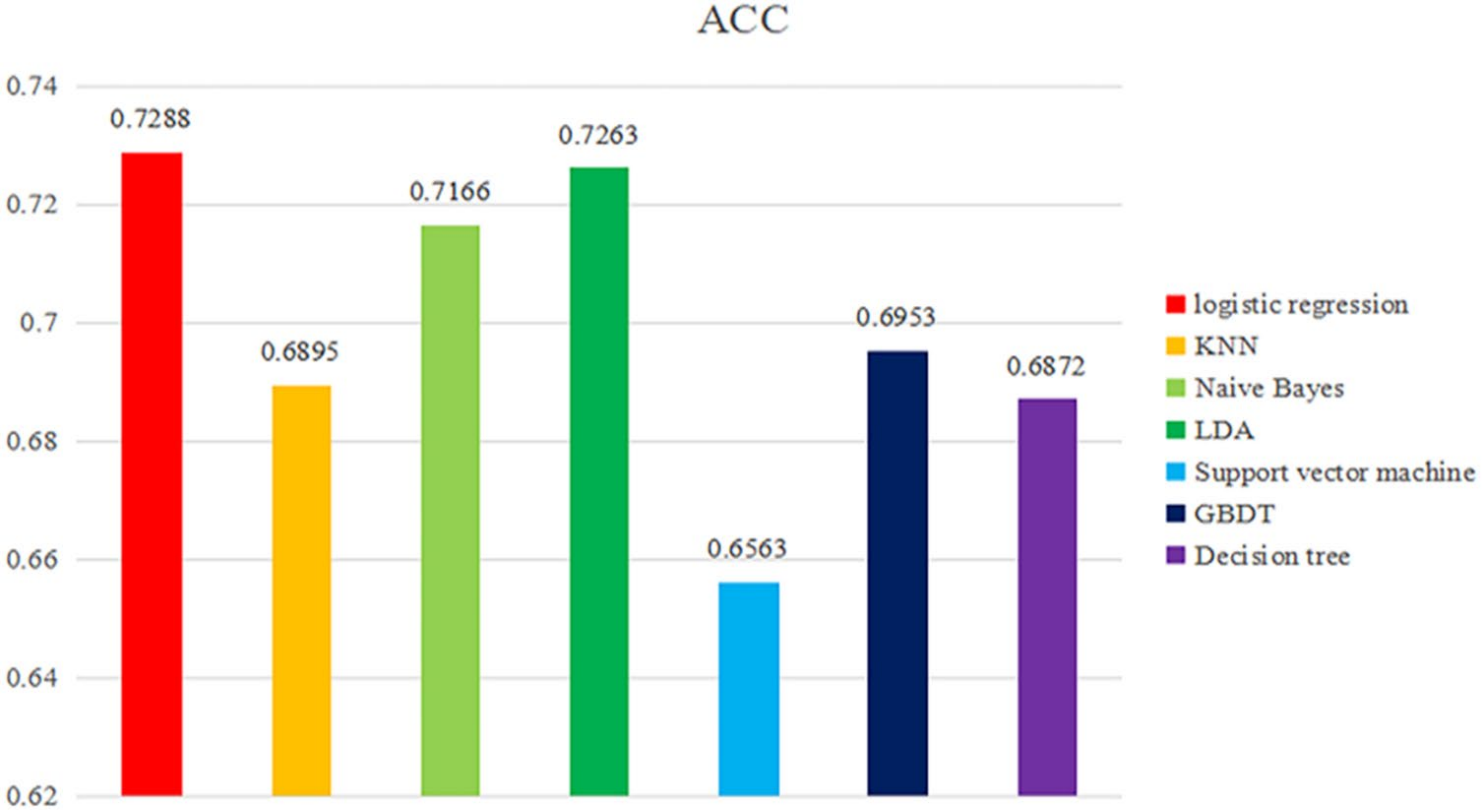
Statistical chart of prediction accuracy of different machine learning algorithms.

Figure 5 shows the prediction accuracy of the model built by the machine learning algorithm. The accuracy rate of model prediction is an index used to evaluate the level of classification models, mainly refers to the ratio of the accurate number of model prediction to the total number. By comparing the prediction accuracy of the model built by different machine learning algorithms. The result show that among the seven classification machine learning algorithms, the accuracy of logical regression is 0.7288, followed by LDA algorithm, and the prediction accuracy is 0.7263. The naive Bayesian classification algorithm ranks third in prediction accuracy. The prediction accuracy of KNN algorithm is 0.6895, the prediction accuracy of support vector machine is 0.6563, the prediction accuracy of GBDT algorithm is 0.6953, and the prediction accuracy of decision tree algorithm is 0.6897. The prediction accuracy of KNN algorithm, support vector machine algorithm, GBDT algorithm and decision tree algorithm is below 70%. Therefore, we believe that the logical regression classification algorithm has the highest accuracy and can best show the prediction of college students’ sports behavior (physical exercise level) based on the combined factors of sports autonomy and sports learning interest in this study.

**Figure 6:**
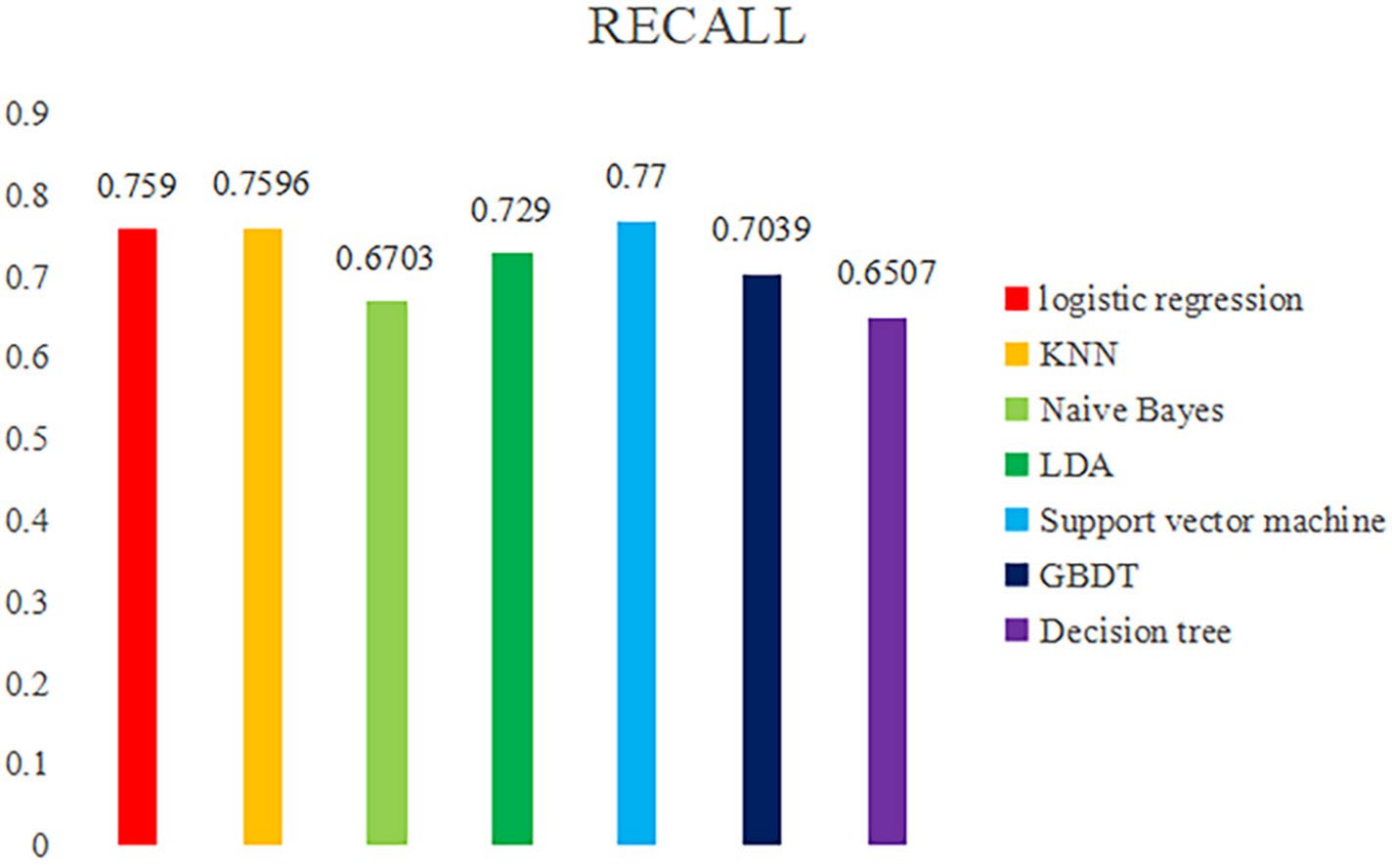
Statistical chart of recall rates predicted by different machine learning algorithms.

Figure 6 is the statistical result of recall rate of machine learning algorithm model in 7, which is used as the basis for judging the overall effect of different machine learning models. Recall rate is the probability of the sample predicted to be 1 in the actual sample of 1 for the original sample. In this study, it mainly refers to the probability of predicting college students’ sports behavior (physical exercise level) according to the display status, and the prediction result is consistent with the original data result. The recall rates of the prediction models built by seven different machine learning algorithms are counted and compared. Among the seven different algorithms, we can find that the highest recall rate is the support vector machine algorithm, with a recall rate of 0.77, followed by the KNN algorithm, with a recall rate of 0.7596, the logical regression algorithm with a recall rate of 0.759, and the other four recall rates are lower than 0.75, which will not be considered in this study.

**Figure 7:**
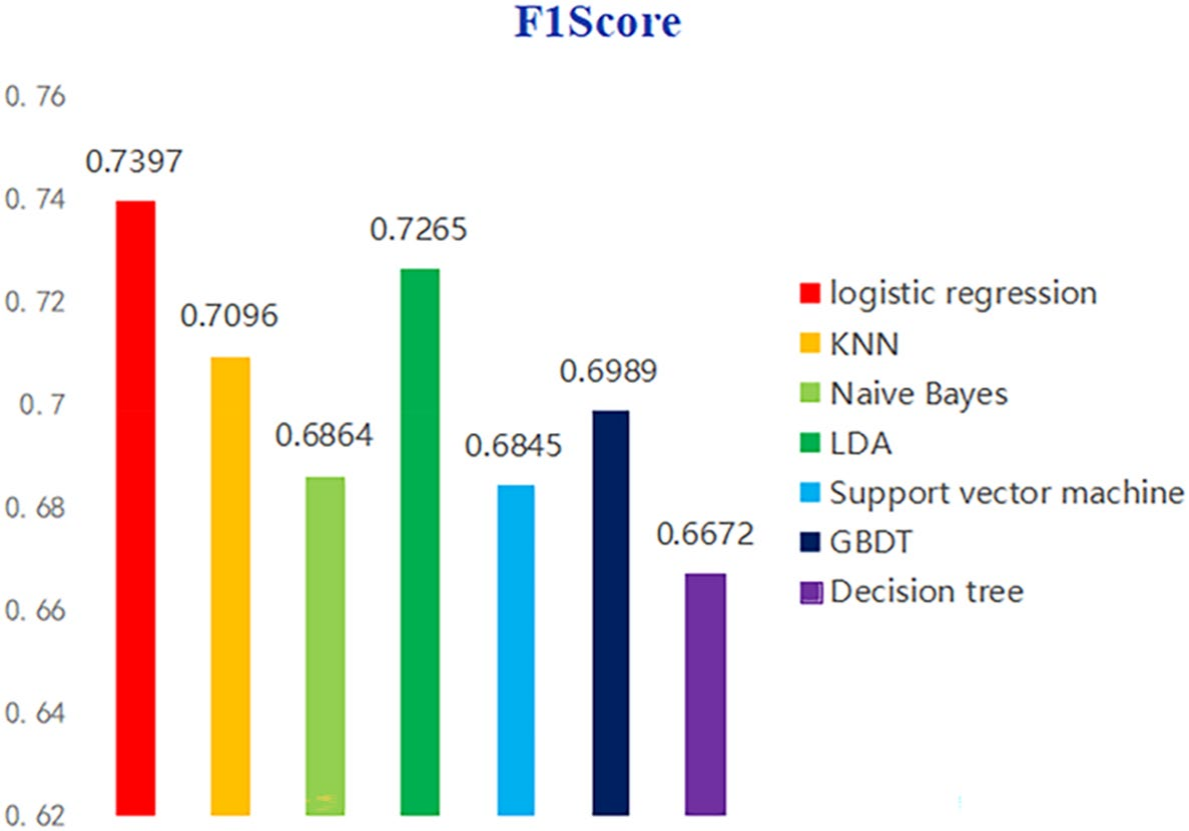
Statistical chart of F1 scores predicted by different machine learning algorithms.

Figure 7 shows the F scores of seven machine learning algorithms. Compare the F1 scores to determine the overall effect of different prediction models. F1 score is an important indicator to evaluate the quality of the model. It is a comprehensive evaluation result based on the accuracy and recall rate. In the evaluation of the model, we hope that the tested model should have both a high level of accuracy and a high level of recall, but the need for both indicators to be high is contradictory. Therefore, an appropriate threshold point is an important means to ensure F1 score. Then determine the threshold point according to the needs of the actual situation, corresponding to the equilibrium point of the discovery accuracy rate and the recall rate, and then define it as F1 score, so that both reach the highest point. The results show that the F1 score of logistic regression is 0.7397, the F1 score of naive Bayes is 0.7265, the F1 score of KNN is 0.7096, and the F1 score of the other four algorithms is lower than 0.7. Therefore, according to the F1 score, the logistic regression model has the highest F1 score in predicting college students’ sports behavior (physical exercise level), which meets the research needs.

**Figure 8:**
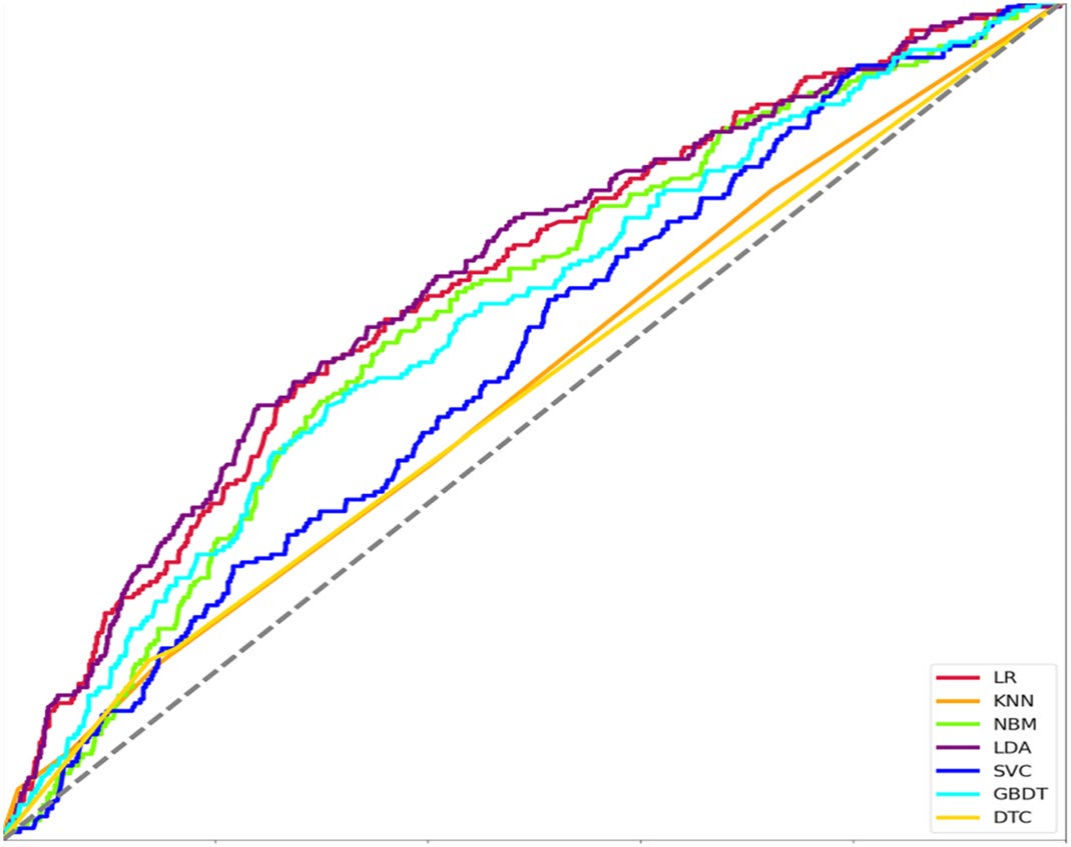
ROC curves of different machine learning algorithms.

Figure 8 is the ROC curve of seven machine learning algorithms, which shows the low quality of prediction models built by different machine learning algorithms. The ROC curve is drawn by traversing all thresholds. The steeper the ROC curve, the higher the model level. The area of ROC curve is used to judge the quality of the model. The data results show that the ranking of the advantages and disadvantages of the model is LDA > LR > NBM > GBDT > SVC > KNN > DTC. Linear discriminant analysis algorithm has the highest model performance, followed by logical regression model algorithm, and the model has the highest superiority.

**Figure 9:**
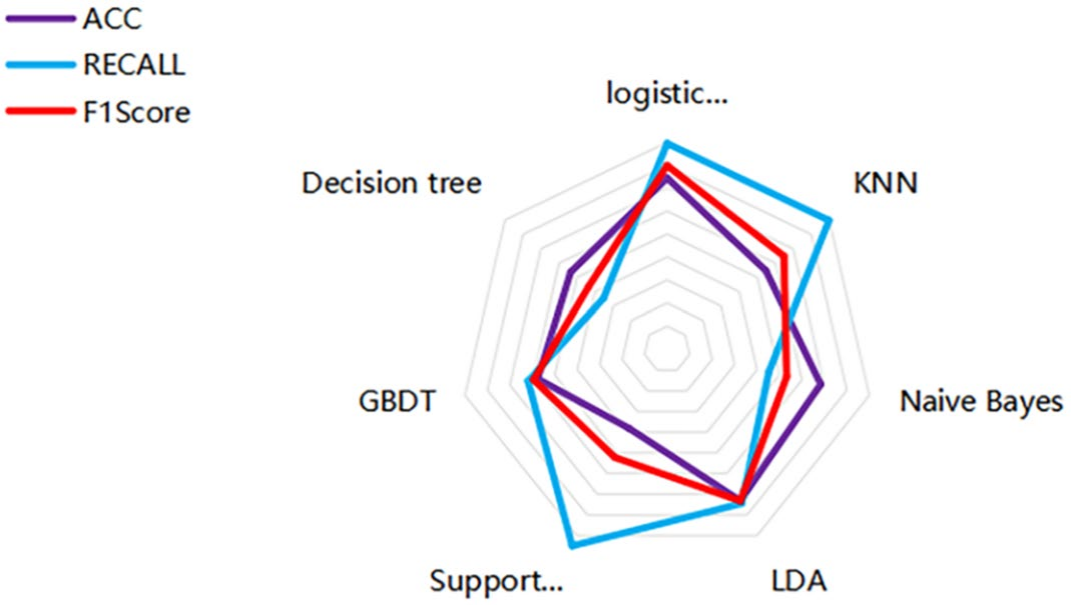
Radar chart of overall evaluation level of different machine learning algorithms.

Table 1 shows the overall statistics of the prediction accuracy, recall rate and F1 score of the prediction models built by the seven machine learning algorithms. Figure 9 shows the radar chart built based on the data in Table 1, which is used to evaluate the overall effect of the prediction models built by the seven machine learning algorithms. The statistical results of the accuracy, recall and F1 scores of the prediction models constructed by different machine learning algorithms, while Fig. 7 is a radar chart constructed based on the accuracy, recall and F1 scores of different prediction models to evaluate the overall effect of the prediction model as a whole. The overall results show that the recall rate of SVM algorithm is the highest, but the accuracy rate and F1 score are low, the recall rate of KNN algorithm is high, and the accuracy rate and F1 score are relatively low, while the accuracy rate, recall rate and F1 score of logistic regression algorithm are the highest. The accuracy, recall and F1 scores of decision tree, naive Bayes, GBDT and LDA algorithms are relatively low. The above chart makes a collective comparison of the accuracy, recall and F1 scores of the seven algorithm models, and determines which algorithm model has the most predictive value through the comprehensive evaluation of three dimensions of indicators. This study points out that the logical regression model is the best among the models to predict college students’ sports behavior (physical exercise level) based on their personal situation, sports autonomy and sports learning.

**Table 1:** Comparative analysis of prediction model differences under different machine learning algorithms.

	ACC	RECALL	F1 score
Logistic regression	0.7288	0.7590	0.7397
KNN	0.6895	0.7596	0.7096
Naive Bayes	0.7166	0.6703	0.6864
LDA	0.7263	0.7290	0.7265
Support vector machine	0.6563	0.7700	0.6845
GBDT	0.6953	0.7039	0.6989
Number of decisions	0.6872	0.6507	0.6672

## Discussion

The research and mining of relevant data in the field of education is an important measure for the integration of machine learning and education. For this new field, it has the characteristics of multi-disciplinary, multi-level, multi-accuracy and situational and semantic. This field covers three fields of education, computer science and statistics, and is the integrated development trend of precision education. Data mining in the field of teaching management and psychology can provide different dimensions of help to students, teachers and education managers. It can enable students to understand their learning deficiencies in real time and adjust learning strategies in time, so as to improve their future performance and conduct more efficient learning. For teachers, according to the predicted and feedback information, teachers can timely understand students’ learning obstacles, adjust teaching methods, customer service learning disability, and improve students’ performance. Managers can make use of the research results in this field to better help teachers and students. Therefore, machine learning will serve and educate more widely in the field of education in the future and serve the development of people. From the prediction accuracy rate of each model, the accuracy rate of the logical regression algorithm is the highest, reaching 72.88%. Because the characteristic variables selected by the research are sports learning interest and sports autonomy, which are potential variables of pedagogy, there are still other factors interfering with their influence on sports behavior (physical exercise level)^31^. The reasons why logistic regression performs well in sports learning interest, sports autonomy support and basic information may include: suitable for data characteristics and patterns, more adaptation to high dimensional characteristics, ability to deal with small samples and category imbalance, and simple, efficient and easy to explain. These features allow the logistic regression to achieve the highest accuracy in this task. This also leads to the prediction accuracy of various algorithms staying at about 70%. In terms of F1 score, the prediction of logistic regression algorithm is still the highest, so it has a significant predictive role in predicting college students’ sports behavior (physical exercise grade) based on joint factors, The prediction model built by the logistic regression algorithm can accurately predict the level of college students’ sports behavior (physical exercise level)^32^. From the analysis of ROC curve results, the distribution and characteristics of the data have an important impact on the model performance. LDA and LR are more suitable for processing some types of data, so they show high model performance in terms of sports learning interest, sports autonomy support and basic information. Although the more training characteristics of college students collected in the study, the higher the effective information that the training model can obtain, because college students have more low-level sports behaviors (physical exercise levels), the proportion of 9:1 was selected when the training set and test set were allocated. At the same time, more potential factors should be considered for modeling in the later prediction model, and the relationship between dominant factors and potential factors should be strengthened.

## Conclusion

Based on the combination of college students’ interest in sports learning and sports autonomy, this study established a machine learning model to predict college students’ sports behavior (physical exercise level), and compared the models constructed by seven algorithms. The research results show that the logistic regression model based on college students’ interest in sports learning and sports autonomy can effectively predict college students’ sports behavior (physical exercise level). Compared with the model constructed by KNN, naive Bayes, LDA, support vector machine, GBDT and decision tree algorithm, the logistic regression model has the highest F1 score and the most obvious prediction accuracy.

## Data Availability

The data used to support the findings of this study are available from the corresponding author upon request.
